# Comparison of Larval and Adult *Drosophila* Astrocytes Reveals Stage-Specific Gene Expression Profiles

**DOI:** 10.1534/g3.114.016162

**Published:** 2015-02-04

**Authors:** Yanmei Huang, Fanny S. Ng, F. Rob Jackson

**Affiliations:** Department of Neuroscience, Sackler School of Graduate Biomedical Sciences, Tufts University School of Medicine, Boston, Massachusetts 02111

**Keywords:** *Drosophila*, astrocyte, translational profiling, vesicle trafficking and secretion

## Abstract

The analysis of adult astrocyte glial cells has revealed a remarkable heterogeneity with regard to morphology, molecular signature, and physiology. A key question in glial biology is how such heterogeneity arises during brain development. One approach to this question is to identify genes with differential astrocyte expression during development; certain genes expressed later in neural development may contribute to astrocyte differentiation. We have utilized the *Drosophila* model and Translating Ribosome Affinity Purification (TRAP)-RNA-seq methods to derive the genome-wide expression profile of *Drosophila* larval astrocyte-like cells (hereafter referred to as astrocytes) for the first time. These studies identified hundreds of larval astrocyte-enriched genes that encode proteins important for metabolism, energy production, and protein synthesis, consistent with the known role of astrocytes in the metabolic support of neurons. Comparison of the larval profile with that observed for adults has identified genes with astrocyte-enriched expression specific to adulthood. These include genes important for metabolism and energy production, translation, chromatin modification, protein glycosylation, neuropeptide signaling, immune responses, vesicle-mediated trafficking or secretion, and the regulation of behavior. Among these functional classes, the expression of genes important for chromatin modification and vesicle-mediated trafficking or secretion is overrepresented in adult astrocytes based on Gene Ontology analysis. Certain genes with selective adult enrichment may mediate functions specific to this stage or may be important for the differentiation or maintenance of adult astrocytes, with the latter perhaps contributing to population heterogeneity.

Glial cells represent an essential cell type in the mammalian and *Drosophila* nervous systems. In the mammalian brain, distinct glial cell classes function in neural development, CNS metabolism, ionic homeostasis (blood–brain barrier function), neuronal excitability, responses to drugs or injury, and behavior (Brown and Ransom 2007; Clasadonte *et al.* 2013; Fields 2006; Halassa *et al.* 2009; Haydon *et al.* 2009; Haydon and Carmignoto 2006; Panatier *et al.* 2006). Similarly, the *Drosophila* nervous system contains multiple classes of glial cells that perform related functions (Awasaki *et al.* 2008; Danjo *et al.* 2011; Edenfeld *et al.* 2005; Edwards and Meinertzhagen 2010; Haydon *et al.* 2009; Jackson 2011; Jackson and Haydon 2008; Jackson *et al.* 2014; Limmer *et al.* 2014; Stork *et al.* 2012; Zwarts *et al.* 2014). Among the different types of glial cells, the astrocyte class has a conserved form in *Drosophila* and mammals, and certain studies have suggested functional similarities (Danjo *et al.* 2011; Rival *et al.* 2006; Rival *et al.* 2004; Stork *et al.* 2014). Our recent expression profiling studies have documented conserved molecular signatures for mouse and *Drosophila* astrocytes (S. Ng, Y. Huang, L. Morel, F. Ng, M. Tolman, S. Sengupta, L. Iyer, F. Jackson, Y. Yang, unpublished data), indicating that this cell type has similar functions in flies and mammals.

Whereas diversity among neuronal populations of the brain is well-understood (Klausberger and Somogyi 2008), little is known about the origins of astrocyte subtypes. However, there is accumulating evidence for heterogeneity among astrocyte populations from studies using insect and mammalian models, although the evidence is more compelling for the mammalian brain (Zhang and Barres 2010). For example, it is known that astrocytes from distinct mammalian brain regions and developmental stages differ with regard to their origin, morphology, molecular signature, physiology, and function. In the past two decades, studies have documented differences in the expression of neuropeptides, ion channels, receptors, transporters, and even glial fibrillary acidic protein (GFAP), once thought to be a canonical marker for differentiated astrocytes (Zhang and Barres 2010). More recent studies using single-cell profiling techniques have documented astrocyte subtypes based on gene expression profiles (Rusnakova *et al.* 2013; Stahlberg *et al.* 2011), with molecular differences including glutamate and GABA receptor composition (Hoft *et al.* 2014) and the expression of gap junction isoforms (connexins) (Griemsmann *et al.* 2014). However, the underlying molecular basis for the development of astrocyte heterogeneity is unknown.

In recent collaborative studies, we used Translating Ribosome Affinity Purification (TRAP) methods to perform expression profiling of *Drosophila* adult and mouse glial astrocytes (S. Ng, Y. Huang, L. Morel, F. Ng, M. Tolman, S. Sengupta, L. Iyer, F. Jackson, Y. Yang, unpublished data). We have now extended this analysis by examining astrocytes of the *Drosophila* larval nervous system with the intent of defining gene expression differences between the two developmental stages. Studies described here have identified 400 genes with enriched expression in adult but not larval astrocytes. These genes are likely to encode proteins important for adult-specific functions or the differentiation/maintenance of adult astrocyte phenotypes.

## Materials and Methods

### *Drosophila* strains and crosses

To generate larvae expressing a tagged ribosome, males homozygous for an *alrm-Gal4* transgene were crossed to homozygous *UAS-EGFP-L10a* females. Gal4 enhancer-trap strains NP5633 and 46117 were obtained from the Kyoto stock collection and Janelia Farm, respectively. NP5633 is just downstream of CG14141, whereas 46117 is in or near the gene encoding the GABA transporter (GAT). Flies were reared at 25° in a light/dark cycle consisting of 12 hr of light and 12 hr of dark (LD 12:12) until the third-instar larval stage. All strains and crosses were raised on our standard laboratory medium consisting of cornmeal, agar, brewer’s yeast, dextrose, sucrose, and wheat germ.

### Affinity purification of translating RNAs from larval astrocytes

Nervous systems were manually dissected from *alrm-Gal4*; *UAS-EGFP-L10a* third-instar larvae between ZT0 and ZT2, washed in phosphate-buffered saline (PBS), and stored at −80° until the time of TRAP analysis. In a typical TRAP experiment, approximately 200 hand-dissected preparations were homogenized and the tissue lysate was cleared and processed as described (Huang *et al.* 2013). One-tenth of the lysate was retained for extraction of total RNA, and the remainder was used for TRAP. Our TRAP procedure has been described in previous publications (Huang *et al.* 2013, 2014). It utilizes a high-affinity GFP antibody for immunoprecipitation (IP) of EGFP-L10a/RNA complexes (19C8 from Monoclonal Antibody Core Facility, Sloan Kettering). Similar to our published work, we added 20 μg of anti-GFP to 1 mg of beads to form the antibody–bead complex prior to the IP. Typically, approximately 30 ng of RNA was recovered by TRAP from 200 larval nervous systems. RNA from several TRAP experiments was pooled to obtain three independent samples of approximately 200 ng each. Similarly, we prepared three independent total RNA samples, also of approximately 200 ng each. These TRAP and total RNA samples were used to construct RNA-seq libraries using the Illumina TruSeq RNA kit (v 2) following a standard protocol provided by the manufacturer.

### RNA-seq and data analysis

RNA-seq libraries were analyzed on an Illumina HiSequation 2000 sequencer at Tufts University Core Facility. Sequence reads were obtained and their quality was analyzed using the quality control metrics provided by the FastQC pipeline (http://www.bioinformatics.babraham.ac.uk/projects/fastqc/). We obtained, on average, 34.5 million high-quality 100-base reads for each of the 6 samples (after removing low-quality reads), and an average of 92.3% of the high-quality reads could be mapped to the *Drosophila* 5.22 reference genome using Tophat2 (v 2.0.8) and Bowtie2 (v 2.1.0) (Langmead and Salzberg 2012; Trapnell *et al.* 2009). Two alternative methods were used to identify RNAs enriched in the TRAP samples compared with total RNA samples. One used the Cufflinks and Cuffdiff programs (Trapnell *et al.* 2013) to identify differentially expressed genes. The second method used HTseq-count (http://www-huber.embl.de/users/anders/HTSeq/doc/overview.html) to obtain direct counts of aligned reads, which were then analyzed using DEseq (Anders and Huber 2010) to define differentially expressed genes (*i.e.*, enriched in TRAP RNA *vs.* total RNA samples). For both analyses, the dispersion method was set to “per condition.” Genes identified by both programs were accepted as enriched in the TRAP RNA samples. The gene association table from FlyBase (October 2014) was used to derive Gene Ontology (GO) terms. The GO Toolset of the Lewis-Sigler Institute Bioinformatics Group (Princeton University) was used for certain analyses.

For comparison of fly and mouse glial-expressed genes, we used normalized expression values from published mouse cortical astrocyte, oligodendrocyte, and Bergmann glia RNA microarray samples (Doyle *et al.* 2008) (NCBI GEO series GSE13379). For each gene, we calculated the ratio of astrocyte or oligodendrocyte expression relative to the average expression in the other two cell types. A fold change of 1.2 was used as a cutoff to select genes enriched in each of the three cell types. The mapping of mouse and fly homologs was performed using BioMart (http://biomart.org), considering only the one-to-one homologs. Custom R-scripts were used to merge the different datasets.

### Immunohistochemistry and imaging

Antibody staining of hand-dissected nervous systems was performed as previously described (Ng *et al.* 2011). Monoclonal antibodies recognizing Repo (University of Iowa Hybridoma Center) and GFP (19C8, Sloan Kettering) were used at dilutions of 1/500 and 1/1000, respectively. Optical sections of 1.5 υm were acquired from nervous system whole mounts using a Leica SP2 AOBS microscope.

## Results

### Larval nervous system TRAP analysis

**Figure 1:**
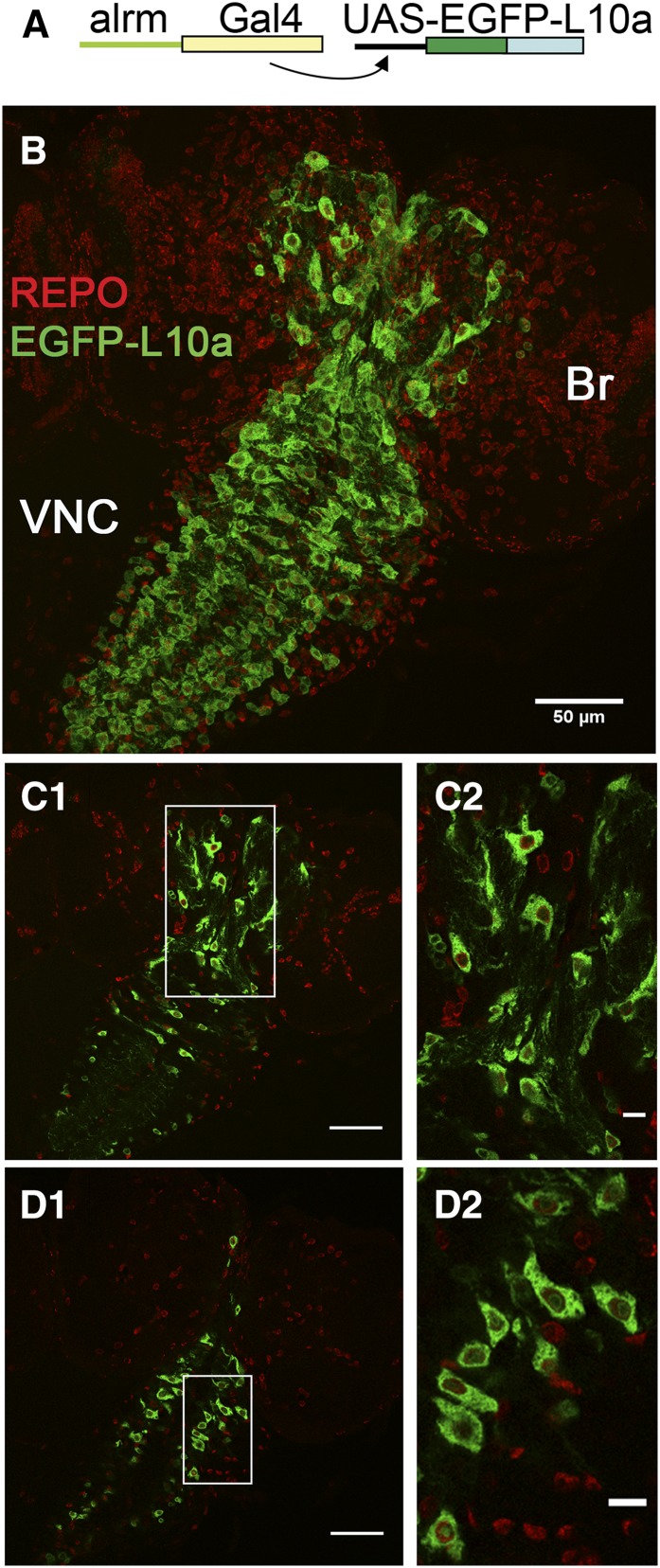
Expression of EGFP-L10a in larval nervous system astrocytes. (A) The *alrm-Gal4* driver was used to limit expression to astrocytes. (B) Low magnification view of the entire larval brain (Br) and ventral nerve cord (VNC) showing alrm-driven expression of EGFP-L10a in both regions. (C1 and C2) View of EGFP-L10a expression in brain astrocytes illustrating cytoplasmic localization of the protein. (D1 and D2) EGFP-L10a expression in the cytoplasm of VNC astrocytes. Green, EGFP-L10a; red, Repo (a glial nuclear marker); size bars = 50 μm in B, C1, and D1, and 10 μm in C2 and D2. The image in (B) represents a Z stack of optical sections representing the whole nervous system. Other images of this figure are 1.5-μm optical sections.

We performed larval TRAP profiling using a previously described astrocyte Gal4 driver strain (*alrm-Gal4*) (Doherty *et al.* 2009). The *alrm-Gal4* driver was combined with a *Drosophila* transgene encoding a tagged large ribosomal subunit (*UAS-EGFP-L10a*) (Huang *et al.* 2013) to generate flies specifically expressing EGFP-L10a in astrocytes of the larval nervous system (brain and ventral nerve cord; Figure 1A). As shown in Figure 1, EGFP-L10a could be detected in glial cells of the larval brain lobes (Figure 1, B and C) and ventral nerve cord (Figure 1, B and D). In both locations, EGFP-L10a was observed to be cytoplasmic, as determined by straining with the Repo nuclear marker (Figure 1, C and D). Expression of alrm-Gal4-driven EGFP-L10a throughout development did not grossly affect astrocyte cell morphology (Figure 1 and data not shown), nor did it affect viability or activity level or cause circadian arrhythmicity for adult animals (Supporting Information, Figure S1).

We performed immunoprecipitation (IP) of ribosome–RNA complexes from hand-dissected larval nervous systems using a high-affinity GFP antibody as previously described (Huang *et al.* 2013). For each of three independent biological replicates, one-tenth of the lysate was used for extraction of total RNA; the remainder was used for anti-GFP immunoprecipitation (TRAP-IP), with subsequent extraction of ribosome-bound RNA. All RNA samples were judged to be of high quality on the basis of Agilent Bioanalyzer analysis. The Illumina TruSeq RNA kit (v 2) was used to generate RNA-seq libraries. Libraries were analyzed by multiplex sequencing on an Illumina HiSequation 2000 instrument.

**Figure 2:**
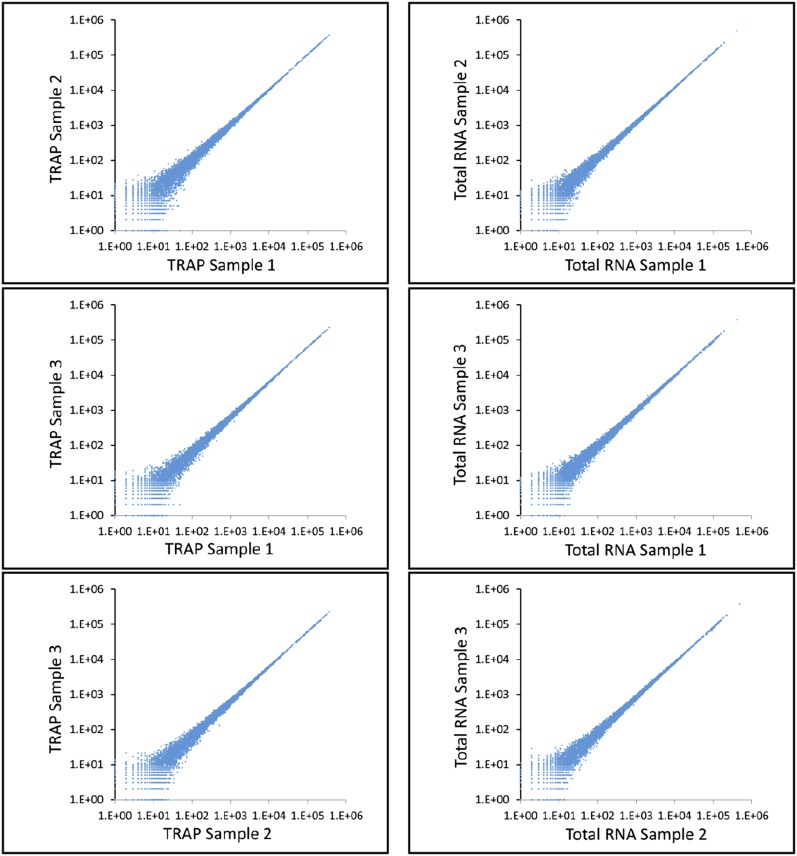
Scatterplots of log10 sequence read values for all genes using HTSeq-count. The X and Y axes are pairwise comparisons of the three TRAP and three total RNA sample replicates to one another.

Three biological replicates were sequenced for the TRAP-IP and total RNA samples. For all TRAP RNA samples, average read numbers ranged from 0 to ∼315,000 for individual genes. Of these astrocyte-expressed genes, 9107 were represented by 20 or more reads in all three samples. For the total RNA samples, the reads averaged 0 to ∼426,000, and 9725 genes were represented by 20 or more reads. All samples had a mean quality score of >30. Figure 2 shows scatter plots indicating the reproducibility of RNA-seq results for the TRAP IP and total RNA replicate samples. A spearman rank correlation analysis indicated that there was greater than a 95% correlation between samples with regard to expression values.

### Genes with enriched expression in larval astrocytes

**Figure 3:**
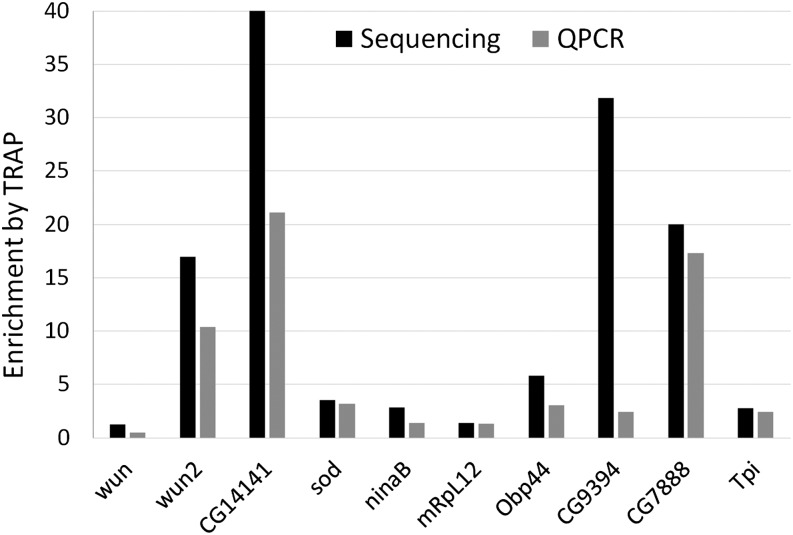
Fold enrichment by TRAP for 10 genes, relative to expression in total RNA. Enrichment was assayed by RNA-seq or QPCR.

We compared the sequencing results from TRAP and total RNA samples to identify those with astrocyte-enriched expression. Two alternative methods (see *Materials and Methods*) revealed a similar number of genes with astrocyte-enriched expression with comparison of TRAP with the total RNA samples: Cuffdiff identified 3750, whereas DESeq identified 3795 genes. Of these, 3186 genes were found to have enriched expression by both programs (Table S1). Note that the values for total RNA and TRAP experiments in Table S1 and all other Supporting Information Tables represent mean values for three independent samples. We examined 10 genes with high, moderate, or low astrocyte-enriched expression—as judged by RNA-seq—using quantitative RT-PCR (QPCR). With the exception of one gene, enrichment values derived from the RNA-seq analysis were similar to those found by QPCR, verifying the sequencing results (Figure 3).

A GO analysis, using the FlyBase gene association table, identified genes required for many biological processes (Table S2). The list of astrocyte-enriched genes includes those known to be (or expected to be) expressed in fly astrocytes. Both the Astrocyte leucine-rich Repeat Molecule (Alrm) and the Reversed Polarity (Repo) transcription factor, which drives glial differentiation, show enriched expression, although we note that Repo is expressed in most glia of the brain. Similarly, the EAAT1 (mammalian GLT1) glutamate transporter, the GABA transporter (GAT), GABA transaminase, Glutamine Synthetases (GS2 and GS1-like), and Dopamine acetyltransferase (Dat)—all with known glial neurotransmitter recycling functions—exhibit astrocyte enrichment. Ebony, a glial β-alanyl-amine synthase that is required for aminergic neurotransmitter recycling, vision, and circadian rhythm (Richardt *et al.* 2003; Suh and Jackson 2007), is also enriched in astrocytes. Astrocyte enrichment for these genes, as assayed by TRAP-RNA-seq, is a validation of our profiling approach.

In addition to expected genes, our analysis revealed several other interesting classes of astrocyte-enriched genes. Not surprisingly, hundreds of larval astrocyte-enriched genes encode proteins important for metabolism, energy production, and protein synthesis, consistent with the known role of astrocytes in the metabolic support of neurons (Brown and Ransom 2007). Many genes, for example, have roles in metabolism, catabolism, cellular respiration, or ATP generation (Table S2). More than 200 genes encode components of protein synthesis, including factors mediating translational initiation, elongation, or termination and the assembly of cytoplasmic or mitochondrial ribosomes (highlighted in yellow in Table S2). This result speaks to the importance of protein synthesis for CNS growth and differentiation. Interestingly, there are more than 60 genes that mediate aspects of vesicle transport, fusion, and recycling (Table S2, blue), with a number of genes encoding factors required for synaptic vesicle exocytosis and endocytosis (Table S2, green). For example, Rop/Munc18, a sec1 homolog that regulates neuronal synaptic vesicle fusion by interaction with syntaxin, is enriched in larval and adult astrocytes (next section), as are eight different syntaxins. Several genes with enriched expression are known to be required for immune/defense responses (*Iap2*, *eiger*, *wun*, *wun2*). Finally, hundreds of genes encode proteins involved with some aspect of nervous system development or function (gray in Table S2). A number of the latter proteins are secreted factors, including eiger [tumor necrosis factor (TNF)], CG34445 (TNF-like), CG14141, and CG7607 (both small Ig-domain proteins). Of these GO categories, metabolism, energy production, vesicle transport, and translation are significantly overrepresented (*P* < 0.01) when the gene list is analyzed using the GO Toolset developed by the Bioinformatics Group at the Lewis-Sigler Institute (Princeton University) in combination with the FlyBase association table.

**Figure 4:**
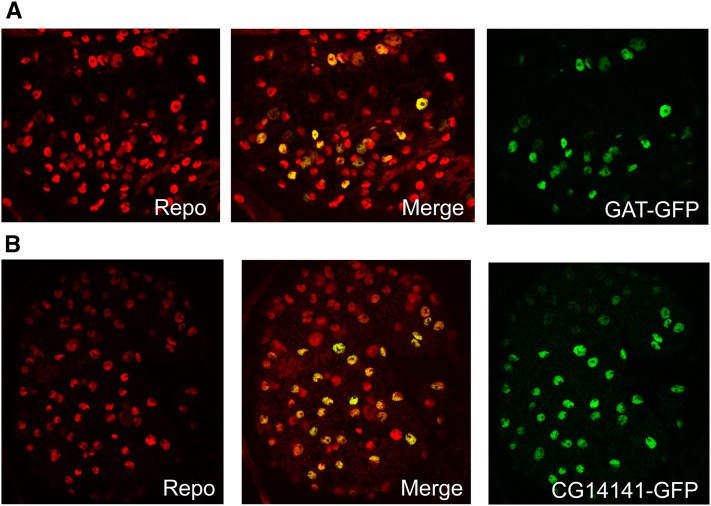
Enhancer trap-driven GFP expression for GAT and CG14141. Strains NP5633 (Kyoto stock collection) and 46117 (Janelia Farm) with Gal4 insertions near CG14141 and in or near GAT were used to drive nuclear GFP. Brains were stained with anti-GFP to detect enhancer trap expression and anti-Repo to reveal all glial cells. As shown in the merge images, both Gal4 drivers express in a subset of Repo-positive glial cells (yellow), consistent with expression in astrocytes.

As already mentioned, many of these genes are known to be expressed in fly astrocytes, based on previous studies. Making use of enhancer trap Gal4 insertions in or near the genes encoding GAT (known astrocyte expression) and a novel enriched factor (CG14141), we examined the expression pattern relative to Repo, a pan-glial factor. Both genes are expressed in a subset of Repo-positive glial cells of the larval brain lobes, consistent with astrocyte expression (Figure 4).

### Genes with astrocyte expression in both larvae and adults

Comparison of larval and adult TRAP results identified 836 genes with enriched expression at both developmental stages (Table S3). This value probably represents an underestimate of common gene number as the adult RNA-seq experiments had reduced sequencing depth compared with the larval experiments (see next section). Similar to larval astrocyte-enriched genes, many of these “common” genes encode proteins important for metabolism, energy production, translation, vesicle-mediated transport and exocytosis, and nervous system development (Table S4). Common genes with highest enrichment encode a homeodomain transcription factor (CG34367), a Glutathione transferase (*GstD3*), and of course the Alrm protein, which is expected to be enriched in *alrm-Gal4 > EGFP-L10a* nervous systems (Table 1). In addition, two relatively small Ig domain-containing proteins and a TNFα-like protein exhibit astrocyte enrichment at both stages; enrichment of one of the Ig domain proteins (CG14141) was validated by QPCR (Figure 3). Both Ig domain proteins have a nervous system–specific pattern of expression (FlyAtlas) (Chintapalli *et al.* 2007) and are predicted to be secreted (Vogel *et al.* 2003). Thus, they may serve intercellular signaling functions in the larval and adult nervous system.

**Table 1:** Genes with the highest astrocyte-enriched expression in both larvae and adults

**Gene**	**Function**	**Expression**	**log2 Enrichment**
CG34367	Homeo domain transcription factor	Brain and testis	4.31
alrm	Leucine-rich molecule	Brain and eye	4.07
wun2	Lipid phosphatase	Brain and other tissues	3.64
GstD3	Glutathione transferase	Brain and other tissues	3.55
Obp44a	Odorant binding	Brain and testis	3.53
CG1537	Unknown	Brain and other tissues	3.51
CG34445	Tumor necrosis factor (TNF) α–like	No brain expression data	3.46
CG1552	Unknown	Brain-specific	3.42
CG14141	Ig domain protein	Brain and eye	3.36
CG33958	Guanylate cyclase	No brain expression data	3.33
CG12926	Vitamin transporter	Brain and other tissues	3.16
CG9394	Phosphodiesterase	Brain and other tissues	3.08
CG1545	Unknown	Brain-specific	3.00
CG15209	Unknown	Brain and other tissues	3.00
CG7607	Ig domain protein	Brain and eye	2.90

### Genes with enriched expression selectively in larvae or adults

Our RNA-seq analysis identified many genes that appeared to have enriched expression in larvae but not adults (Table S5). However, the larval data were obtained from samples containing the brain lobes and ventral nerve cord (thoracic and abdominal ganglia), whereas the adult samples represented only the brain (minus the thoracic and abdominal ganglia). In addition, the larval data are of much higher sequencing depth (averaging 34.5 million reads per sample compared with 15.6 million reads per sample for adult astrocytes). Thus, there is greater statistical power for the larval profiling, which might result in a higher number of genes with significantly enriched expression. Thus, genes identified in the larval (but not the adult) dataset may not actually be specific for the larval stage. In contrast, the genes with enriched expression in adult but not larval astrocytes are of greater interest. Four hundred such genes with selective adult astrocyte-enriched expression were identified in our profiling studies (Table S6). They fall into many GO categories, including those important for metabolism, energy production, translation, and nervous system function (Table S7), consistent with the known role of astrocytes in neural support.

The top 15 “adult-specific” genes (Table 2) encode four different protein kinases (CGs 10514, 32195, 11892, and 7135), a neuropeptide signaling molecule (SIFa), an amino acid transporter (CG43693), and a sialic acid synthase (Csas). We note that 9 of these 15 genes (highlighted in blue) are known to have higher expression in the adult *vs.* larval CNS, based on information from FlyAtlas (Chintapalli *et al.* 2007). These genes and others showing adult-specific enrichment may encode proteins with adult-specific functions or factors required for astrocyte differentiation and/or maintenance. The latter function may be influenced by the *Drosophila rau* and *pros* genes, both of which are selectively enriched in adult astrocytes and known to mediate aspects of fly glial cell development or differentiation (Kato *et al.* 2011; Sieglitz *et al.* 2013). Pros is a homeodomain transcription factor, whereas rau is a ras-GTPase binding protein; each has mouse and human homologs.

**Table 2:** Genes with high astrocyte-enriched expression only in adults

**Gene**	**Function**	**Expression**	**log2 Enrichment**
SIFa	Neuropeptide signaling	Brain enriched	3.82
CG10514	protein kinase–like	Brain, MT, and spermatheca	3.76
Hsp67Bc	Chaperone	Brain and MT	3.55
Irk3	Potassium channel	Brain and MT	3.46
Tdc2	Tyrosine decarboxylase	Brain-specific	3.44
CG6465	Peptidase	Brain and other tissues	3.37
CG15201	Unknown	Brain and other tissues	3.22
CG12269	Sterol binding	Brain and eye	3.06
Hsp23	Chaperone	Brain and other tissues	3.01
CG32195,CG7341	Protein kinase-like	Brain and other tissues	2.86
CG11892	Protein kinase-like	Brain, MT, and spermatheca	2.75
CG7135	Protein kinase-like	Brain and eye	2.74
CG43693	Amino acid transporter	No expression available	2.63
CG13670	Insect cuticle	Eye	2.63
Csas	CMP-sialic acid synthetase	Brain and eye	2.61

Blue shading indicates genes with higher expression in the adult than the larval nervous system based on information from FlyAtlas.

Other adult enriched genes encode factors important for mating or courtship behaviors, circadian behavior, learning and memory, immune defense, neuropeptide signaling, and neurotransmission (Table S7). Interestingly, a number of proteins critical for neurosecretion exhibit astrocyte enrichment in adults but not larvae [N-ethylmaleimide-sensitive factor 1 (NSF1), several soluble NSF attachment proteins (SNAPs), a SNAP-associated protein (Snapin), and Ceramidase (CDase)]. Consistent with an adult function for NSF1, which is critical for vesicle-cell membrane fusion, the *NSF1* gene was previously shown to be more highly expressed in adults than in larvae (Pallanck *et al.* 1995). Also of interest, we do not observe evidence for adult astrocyte enrichment of NSF2, an alternative *Drosophila* NSF molecule. This suggests that NSF1, but not NSF2, may function in adult astrocytes as well as neurons.

**Figure 5:**
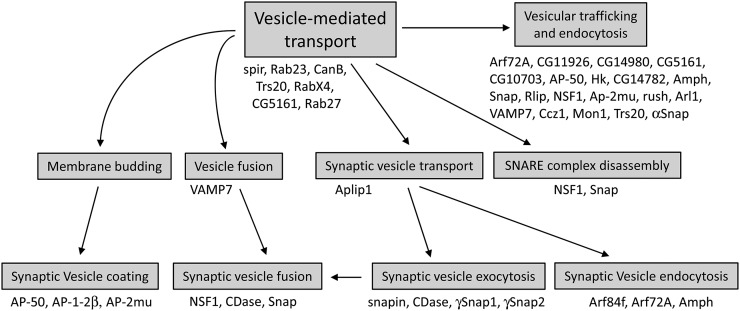
Gene ontology (GO) relationships for adult-enriched genes/proteins involved in vesicle-mediated transport and vesicle secretion. The expression of genes encoding these proteins is overrepresented in adult astrocytes, as indicated by the GO Toolset (Princeton University). Note that this figure does not include other factors, such as Rop, syntaxins, sec homologs, Rab proteins, and others, which exhibit astrocyte-enrichment in both larvae and adults.

To asses GO overrepresentation in the genes with adult-specific enrichment, we again used the GO Toolset (Princeton University). Using a cutoff of *P* < 0.01, we determined that two major GO terms were overrepresented in the adult genes: chromatin modification and vesicle-mediated transport (Table S8). The latter category includes genes important for intracellular transport and neurosecretion. Figure 5 illustrates the relationships between GO categories and genes that fall under vesicle-mediated transport. Many of these genes encode factors important for vesicle secretion. We note that this figure only shows genes found to have adult-specific enrichment. There are many other factors required for vesicular trafficking or secretion that show astrocyte enrichment in larvae and in adults (Table S3 and Table S4). Overrepresentation of this category of genes strongly suggests an important function for astrocyte secretion in the adult fly CNS.

We compared fly astrocyte-enriched genes with the databases of Doyle *et al.* (2008) to identify mouse homologs with astrocyte or oligodendrocyte enrichment (see *Materials and Methods*). The studies of Doyle *et al.* (2008) used the TRAP procedure, combined with microarray analysis, to define mouse genes with glial-enriched expression patterns. Notably, 1476 of the identified larval genes (Table S1) have mouse homologs and 820 of these mouse genes (56%) show enriched expression in astrocytes. Similarly, 248 of 407 mouse genes (61%) with homology to the larval-adult “common” gene set (Table S3) and 59 of 124 mouse genes (49%) homologous to those of Table S6 (“adult-specific” enrichment) show enriched expression in mouse astrocytes. In contrast, the fly astrocyte-enriched gene expression profiles are much less similar to those representing mouse oligodendrocytes (26%, 22%, and 21% for larval *vs.* oligo, larval-adult *vs.* oligo, and adult *vs.* oligo comparisons). These findings lend credence to the use of *Drosophila* as a genetic platform for studying astrocyte functions.

## Discussion

We have derived the genome-wide expression profile for *Drosophila* larval astrocytes using cell type–specific translating RNA isolation coupled with RNA-seq analysis. The studies identified a number of genes known to be expressed in fly astrocytes, providing a validation of our profiling approach. The GO analysis has identified numerous larval and adult astrocyte-enriched genes with functions in cellular metabolism, energy production, translation, intercellular signaling, and nervous system development /function. These are expected GO categories, given the known roles of glial cells in neuronal support. Many genes with astrocyte-enriched expression encode vesicle trafficking and secretion components, and this gene set is significantly overrepresented in larval and in adult astrocytes. These genes are also phylogenetically conserved, with homologs present in mammalian species. Because less is known about transmitter secretion from astrocytes, compared with neuronal secretion mechanisms, the identification of such genes facilitates a genetic analysis of gliotransmission.

Our studies also identified fly genes with adult astrocyte-enriched expression that encode potentially secreted factors, including Ig-domain proteins and neuropeptides (Table 1 and Table 2). Surprisingly, a number of neuropeptide-encoding genes show adult-selective enrichment including *SIFa*, which has been implicated in sleep regulation (Park *et al.* 2014) and circadian output (Cavanaugh *et al.* 2014). To our knowledge, it has not been reported that *SIFa* might be expressed in glial cells. In addition, our studies suggest enrichment for Tdc2 RNA, encoding a neurotransmitter synthetic enzyme, but given the well-characterized expression pattern for this gene, the result may be an artifact of very high neuronal expression and nonspecific IP of the RNA. Studies of astrocyte-enriched secreted factors may yield insights about adult-specific glia-neuron signaling functions.

Many genes with selective enrichment in adult astrocytes have roles in development and differentiation, and more than 20 have transcriptional regulatory functions (Table S6). The latter gene class may be important for astrocyte development or differentiation. In addition, the expression of genes relevant for chromatin modification and other cellular functions (*e.g.*, HDACs) is overrepresented in adult astrocytes. This category includes 15 genes, all of which are expressed in the adult CNS (FlyAtlas), with many having known neural functions. Interestingly, the cytoplasmic deacetylase HDAC6 is enriched in adult astrocytes, and it was recently shown that it deacetylates Bruchpilot (Miskiewicz *et al.* 2014), a component of the neuronal presynaptic density that tethers synaptic vesicles and regulates vesicle release. It is an intriguing idea that HDAC6 has a role in the differentiation of the astrocyte secretion machinery.

The adult-specific enrichment of *Csas*, encoding a cytidine monophosphate (CMP)-sialic acid synthetase, is notable with regard to nervous system and perhaps astrocyte differentiation. This enzyme mediates sialylation, a type of N-linked glycosylation event that adds sialic acid moieties to proteins, and this modification is known to be critical for nervous system function. N-glycosylation of neurotransmitter receptors, ion channels, transporters, and cell adhesion molecules is critical for nervous system development and function, with sialylated N-linked glycans being strong regulators of neural cell adhesion molecule (NCAM) as well as sodium and potassium channels (Scott and Panin 2014). FlyAtlas expression data and a recent analysis of *Drosophila Csas* document a nervous system–specific pattern of expression in fly embryos, larvae, and adults (Chintapalli *et al.* 2007; Islam *et al.* 2013). Islam *et al.* (2013) show that there is a broad pattern of *Csas* expression in the adult CNS that includes glia-containing neuropil regions, although there was no direct indication of glial expression in that study. Null mutants of *Csas* exhibit an adult temperature-sensitive paralysis, similar to that observed for the *Drosophila paralytic* (*para*) and *seizure* (*sei*) channel mutants (Jackson *et al.* 1985; Wang *et al.* 1997; Wu and Ganetzky 1980), and *Csas* mutant phenotypes are enhanced by *para* and suppressed by *sei* alleles (Islam *et al.* 2013). Consistent with this interaction, *Csas* mutants have altered neural transmission at the larval neuromuscular junction (Islam *et al.* 2013), but physiological analysis of adult mutants has not been reported.

*Csas* is enriched in adult, but not larval, astrocytes. In contrast, the *DSiaT* gene, which encodes a sialyltransferase mediating sialylation of ion channels and other factors, is not enriched in adult fly astrocytes (nor apparently even expressed in adult astrocytes according to our RNA-seq data), even though it is thought to act downstream of *Csas* in the same biochemical pathway. Consistent with this result, published studies show a neuron-specific pattern of expression for *DSiaT* in the adult fly nervous system, and genetic interaction analysis suggests the two enzymes may have independent functions (Repnikova *et al.* 2010). Thus, *Csas* and *DSiaT* may selectively function in astrocytes and neurons, respectively, of the adult nervous system. *Csas* function and sialylation may be important for astrocyte differentiation or the maintenance of mature astrocyte phenotypes.

## 

## Supplementary Material

Supporting Information
