# Supplementary material for: Comparison of Larval and Adult Drosophila Astrocytes Reveals Stage-Specific Gene Expression Profiles
Source: G3 (Bethesda). 2015 Feb 4;5(4):551–8. doi: 10.1534/g3.114.016162 (PMC4390571; doi:10.1534/g3.114.016162)
Supplement: Supporting Information [file supp_5_4_551__index.html]

Comparison of Larval and Adult Drosophila Astrocytes Reveals Stage-Specific Gene Expression Profiles — Supporting Information 

# Comparison of Larval and Adult *Drosophila* Astrocytes Reveals Stage-Specific Gene Expression Profiles

## Supporting Information for Huang, Ng, and Jackson, 2015

**Files in this Data Supplement:**

- Supporting Information - Figure S1 and descriptions of Tables S1-S8 (PDF, 221 KB)
- Figure S1 - Representative circadian actograms and correlograms (above) for control flies and those expressing EGFP::L10a in astrocytes (alrm-Gal4>UAS-EGFP::L10a, elavG80). (PDF, 430 KB)
- Table S1 - Genes with astrocyte-enrichment in third instar larval nervous system. In Tables S1, 3, and 5, mean values are shown for sequence reads derived from TRAP or total RNA samples. Also shown are log2 enrichment values (log2 fold change) and the actual fold change for individual genes. (.xlsx, 190 KB)
- Table S2 - Gene Ontology (GO) categories for larval astrocyte-enriched genes. (.xlsx, 129 KB)
- Table S3 - Genes enriched in both larval and adult astrocytes. (.xlsx, 56 KB)
- Table S4 - GO categories for genes with enriched expression in both larval and adult astrocytes. (.xlsx, 54 KB)
- Table S5 - Genes with apparent enrichment only in larval astrocytes. (.xlsx, 138 KB)
- Table S6 - Genes with enrichment only in adult astrocytes. Note that certain sequenced regions were homologous to overlapping genes, and both genes are indicated in column A. (.xlsx, 33 KB)
- Table S7 - GO categories for genes with astrocyte enrichment only in adults. (.xlsx, 40 KB)
- Table S8 - Overrepresented GO categories for adult-selective astrocyte enriched genes. (.xlsx, 11 KB)
