# Supplementary material for: Comparison of Larval and Adult Drosophila Astrocytes Reveals Stage-Specific Gene Expression Profiles
Source: G3 (Bethesda). 2015 Feb 4;5(4):551–8. doi: 10.1534/g3.114.016162 (PMC4390571; doi:10.1534/g3.114.016162)
Supplement: Supporting Information [file supp_g3.114.016162_016162SI.pdf]

G3· Genes | Genomes | Genetics

Comparison of larval and adult *Drosophila* astrocytes reveals stage-specific gene expression profiles

Yanmei Huang [1], Fanny S. Ng and F. Rob Jackson

Department of Neuroscience, Sackler School of Graduate Biomedical Sciences, Tufts University School of Medicine, Boston, MA 02111.
[1] Current address: Bioinformatics and Research Computing, Whitehead Institute for Biomedical Research, Cambridge, MA 02142

Corresponding author: F. Rob Jackson; Department of Neuroscience, Tufts University School of Medicine, 136 Harrison Avenue, Boston, MA 02111
E-mail, rob.jackson@tufts.edu; Tel. 617.636.6752; Fax 617.636.3459

DOI: 10.1534/g3.114.016162

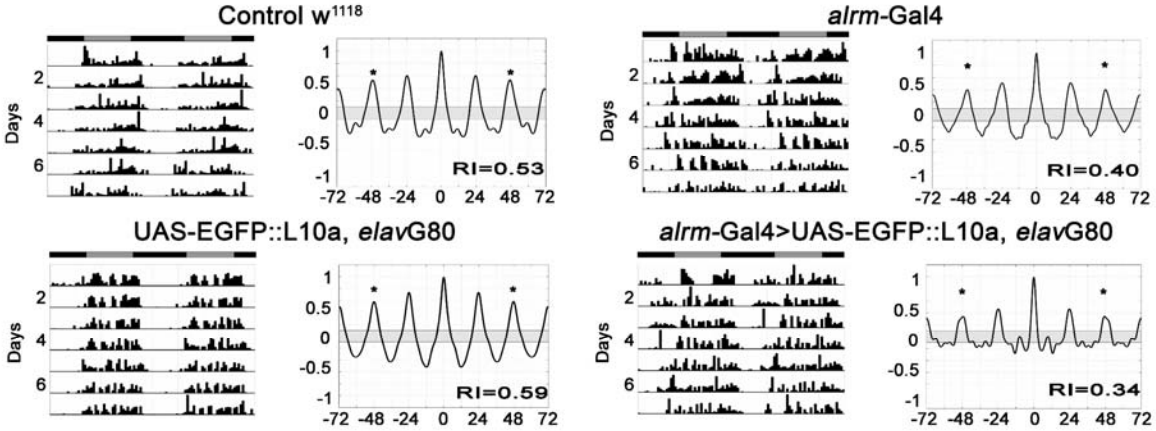

Genotype	LD (4 days at 23°C)				DD (10 days at 23°C)				
	N	Mean activity ± SEM	RI±SEM	%E	N	Mean activity ± SEM	Period ± SEM	RI±SEM	%R
Control w^{1118}	48	22.53±2.31	0.38±0.01	90	43	22.38±2.38	23.67±0.04	0.52±0.01	98
alrm-Gal4	31	30.45±1.73	0.39±0.02	100	31	22.51±1.44	24.30±0.11	0.40±0.02	94
UAS-EGFP::L10a, *elav*G80	32	27.69±1.37	0.41±0.02	94	30	25.20±2.10	23.95±0.06	0.56±0.01	100
*alrm*Gal4> UAS-EGFP::L10a, *elav*G80	64	29.61±1.23	0.35±0.01	97	62	21.24±1.08	24.30±0.11	0.33±0.02*	100

Figure S1 Representative circadian actograms and correlograms (above) for control flies and those expressing EGFP::L10a in astrocytes (alrm-Gal4>UAS-EGFP::L10a, elavG80). Alrm-Gal4>UAS-EGFP::L10a, elavG80 flies carry elavGal80 (elavG80) to prevent expression of EGFP-::L10a in neurons. Each actogram shows 8 days of activity (black histograms) in constant darkness. The correlograms beside each actogram illustrate the robustness of circadian rhythmicity (Rhythmicity Index or RI) with stars indicating statistical significance ($p<0.01$). A table of population statistics (activity level, RI and period) for all genotypes is shown at the bottom of the figure. %E and %R show percent entrainment and percent rhythmicity for the different populations. The star in the table indicates a small but statistically significant difference in RI (0.33 vs 0.40) between alrm-Gal4>UAS-EGFP::L10a, elavG80 and control alrm-Gal4 flies.

Tables S1-S8

Available for download as Excel files at http://www.g3journal.org/lookup/suppl/doi:10.1534/g3.114.016162/-/DC1

Table S1. Genes with astrocyte-enrichment in third instar larval nervous system. In Tables S1, 3, and 5, mean values are shown for sequence reads derived from TRAP or total RNA samples. Also shown are log_2 enrichment values (log2 fold change) and the actual fold change for individual genes.

Table S2. Gene Ontology (GO) categories for larval astrocyte-enriched genes.

Table S3. Genes enriched in both larval and adult astrocytes.

Table S4. GO categories for genes with enriched expression in both larval and adult astrocytes.

Table S5. Genes with apparent enrichment only in larval astrocytes.

Table S6. Genes with enrichment only in adult astrocytes. Note that certain sequenced regions were homologous to overlapping genes, and both genes are indicated in column A.

Table S7. GO categories for genes with astrocyte enrichment only in adults.

Table S8. Overrepresented GO categories for adult-selective astrocyte enriched genes.
