# Supplementary material for: Comparison of Larval and Adult Drosophila Astrocytes Reveals Stage-Specific Gene Expression Profiles
Source: G3 (Bethesda). 2015 Feb 4;5(4):551–8. doi: 10.1534/g3.114.016162 (PMC4390571; doi:10.1534/g3.114.016162)
Supplement: Supporting Information [file supp_g3.114.016162_FigureS1.pdf]

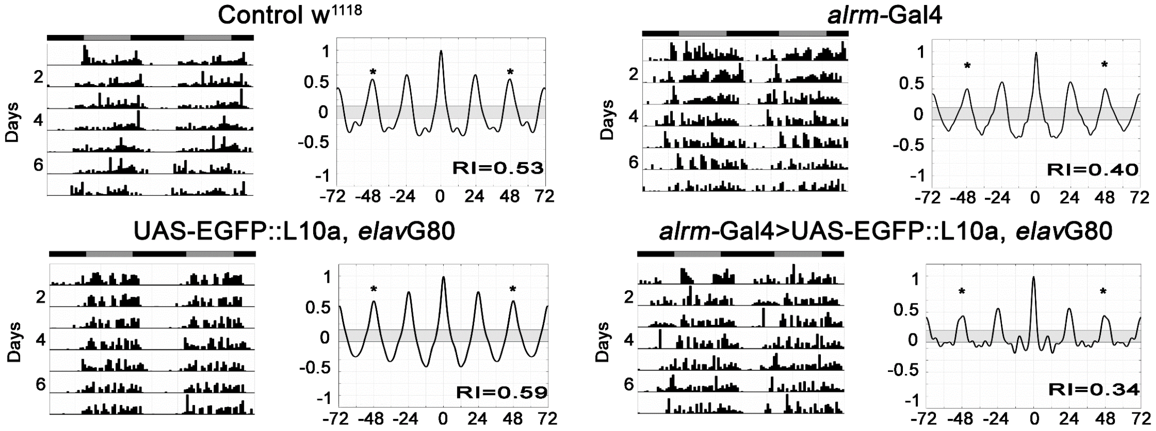

Genotype	LD (4 days at 23°C)				DD (10 days at 23°C)				
	N	Mean activity ± SEM	RI±SEM	%E	N	Mean activity ± SEM	Period ± SEM	RI±SEM	%R
Control w^{1118}	48	22.53±2.31	0.38±0.01	90	43	22.38±2.38	23.67±0.04	0.52±0.01	98
alrm-Gal4	31	30.45±1.73	0.39±0.02	100	31	22.51±1.44	24.30±0.11	0.40±0.02	94
UAS-EGFP::L10a, *elav*G80	32	27.69±1.37	0.41±0.02	94	30	25.20±2.10	23.95±0.06	0.56±0.01	100
*alrm*Gal4> UAS-EGFP::L10a, *elav*G80	64	29.61±1.23	0.35±0.01	97	62	21.24±1.08	24.30±0.11	0.33±0.02*	100

Figure S1 Representative circadian actograms and correlograms (above) for control flies and those expressing EGFP::L10a in astrocytes (alrm-Gal4>UAS-EGFP::L10a, elavG80). Alrm-Gal4>UAS-EGFP::L10a, elavG80 flies carry elavGal80 (elavG80) to prevent expression of EGFP-::L10a in neurons. Each actogram shows 8 days of activity (black histograms) in constant darkness. The correlograms beside each actogram illustrate the robustness of circadian rhythmicity (Rhythmicity Index or RI) with stars indicating statistical significance ($p<0.01$). A table of population statistics (activity level, RI and period) for all genotypes is shown at the bottom of the figure. %E and %R show percent entrainment and percent rhythmicity for the different populations. The star in the table indicates a small but statistically significant difference in RI (0.33 vs 0.40) between alrm-Gal4>UAS-EGFP::L10a, elavG80 and control alrm-Gal4 flies.
